# Can social adversity and mental, physical and oral multimorbidity form a syndemic? A concept and protocol paper

**DOI:** 10.3389/fpsyt.2024.1426054

**Published:** 2025-01-23

**Authors:** Easter Joury, Eliana Nakhleh, Ed Beveridge, Derek Tracy, Ellie Heidari, David Shiers, Silke Vereeken, Emily Peckham, Simon Gilbody, Jayati Das-Munshi, Farida Fortune, Vishal R. Aggarwal, Masuma Mishu, Joseph Firth, Kamaldeep Bhui

**Affiliations:** ^1^ Centre for Dental Public Health and Primary Care, Barts and the London School of Medicine and Dentistry, Queen Mary University of London, London, United Kingdom; ^2^ Barts Health National Health Service (NHS) Trust, London, United Kingdom; ^3^ Homerton College, University of Cambridge, Cambridge, United Kingdom; ^4^ UCLPartners, London, United Kingdom; ^5^ West London National Health Service (NHS) Trust, London, United Kingdom; ^6^ Brunel University Medical School, London, United Kingdom; ^7^ Department of Psychosis Studies, Institute of Psychiatry, Psychology and Neuroscience, King’s College London, London, United Kingdom; ^8^ Division of Psychiatry, Department of Brain Sciences, Imperial College London, London, United Kingdom; ^9^ Faculty of Dentistry, Oral & Craniofacial Sciences King’ College London, London, United Kingdom; ^10^ Department of Sedation and Special Care Dentistry, Guy’s Hospital, London, United Kingdom; ^11^ Greater Manchester Mental Health National Health Service (NHS) Foundation Trust, Manchester, United Kingdom; ^12^ Early Psychosis Unit, University of Manchester, Manchester, United Kingdom; ^13^ University of Keele, Staffordshire, United Kingdom; ^14^ Department of Health Sciences, Hull York Medical School, University of York, York, United Kingdom; ^15^ School of Health Sciences, Bangor University, Gwynedd, United Kingdom; ^16^ Hull York Medical School, University of York, York, United Kingdom; ^17^ Department of Psychological Medicine, Institute of Psychiatry, Psychology, and Neuroscience, King’s College London, London, United Kingdom; ^18^ Centre for Clinical and Diagnostic Oral Sciences, Barts and the London School of Medicine and Dentistry, Queen Mary University of London, London, United Kingdom; ^19^ London Behçet’s Centre, Barts Health London, London, United Kingdom; ^20^ School of Dentistry, University of Leeds, Leeds, United Kingdom; ^21^ Institute of Epidemiology and Health Care, University College London, London, United Kingdom; ^22^ Division of Psychology and Mental Health, University of Manchester, Manchester Academic Health Science Centre, Manchester, United Kingdom; ^23^ Greater Manchester Mental Health National Health Service (NHS) Foundation Trust, Manchester Academic Health Science Centre, Manchester, United Kingdom; ^24^ Department of Psychiatry, Nuffield Department of Primary Care Health Science, University of Oxford, Oxford, United Kingdom; ^25^ Wadham College, University of Oxford, Oxford, United Kingdom; ^26^ East London and Oxford Health National Health Service (NHS) Foundation Trusts, London, United Kingdom; ^27^ World Psychiatric Association (WPA) Collaborating Centre Oxford, Oxford, United Kingdom

**Keywords:** mental disorders, physical conditions, dental diseases, syndemics, conceptualisation, methodology, integrated care, health equity

## Abstract

**Background:**

Clustering mental, physical and oral conditions reduce drastically the life expectancy. These conditions are precipitated and perpetuated by adverse social, economic, environmental, political and healthcare contextual factors, and sustained through bidirectional interactions forming potentially a ‘syndemic’. No previous study has investigated such potential syndemic. Thus, the present project aimed to (i) test for syndemic interactions between social adversity (socioeconomic adversity and traumatic events) and mental, physical and oral multimorbidity using the syndemic theoretical framework; and (ii) determine whether the syndemic relationships vary by age, sex and ethnicity.

**Methods:**

Data from three large-scale population-based databases: UK BioBank, US National Health and Nutrition Examination Survey (NHANES) and the Research with East London Adolescents Community Health Survey (RELACHS) will be analysed. Structural equation modelling (SEM) will be utilised to conceptualise syndemic factors and model complex relationships between directly observed and indirectly observed (latent) variables (syndemic constructs).

**Discussion:**

the syndemic conceptualisation provides a valuable framework to understand health and illness, and hence to better design and deliver effective and cost-effective preventative and curative integrated (syndemic) care to improve patient and population health. Such syndemic care aims to address the social determinants of health, whilst simultaneously managing all interlocked conditions.

## Introduction

The relationship between social adversity and mental, physical and oral multimorbidity is a topical yet under-researched area. A recent critical review proposed that such relationship is likely to form a syndemic (1). The review built its argument on the fact that mental disorders, physical multimorbidity and oral diseases share the same social determinants and have bidirectional relationships that are likely to have synergistic effects in terms premature mortality and excessive burden of morbidity (1–4).

Syndemics are defined as “the aggregation of two or more diseases or other health conditions in a population in which there is some level of deleterious biological or behaviour interface that exacerbates the negative health effects of any or all of the diseases involved” (5). Such adverse disease interactions can occur amongst all types of diseases infectious or non-communicable. They emerge in populations that experience social adversities such as poverty, stigmatisation, systemic discrimination and violence.

Though used interchangeably to describe the co-existence of multiple conditions in any given population, the syndemics concept goes beyond the concepts of comorbidity and multimorbidity. ‘Comorbidity’ describes the co-occurrence of additional conditions alongside a primary ‘index’ condition (6). ‘Multimorbidity’ refers to several conditions that co-exist in tandem but there is no single focus of attention on one condition over the others (7). However, ‘syndemics’ emphasise the adverse interaction between co-occurring diseases (bio-bio interaction) and underscore the role of precipitating and perpetuating adverse social, economic, environmental, political and healthcare contextual factors. These factors cluster with the myriad of diseases and shape their interaction (bio-social interaction) (8). Both types of interaction (bio-bio and bio-social) should exist to form a syndemic. The nature of these interactions should be “synergistic” rather than “additive”; meaning that the produced disease burdens are far more than the sum of disease burdens of each condition or factor alone. A few studies have operationalised the above syndemic concept correctly (8, 9).

**Figure 1:**
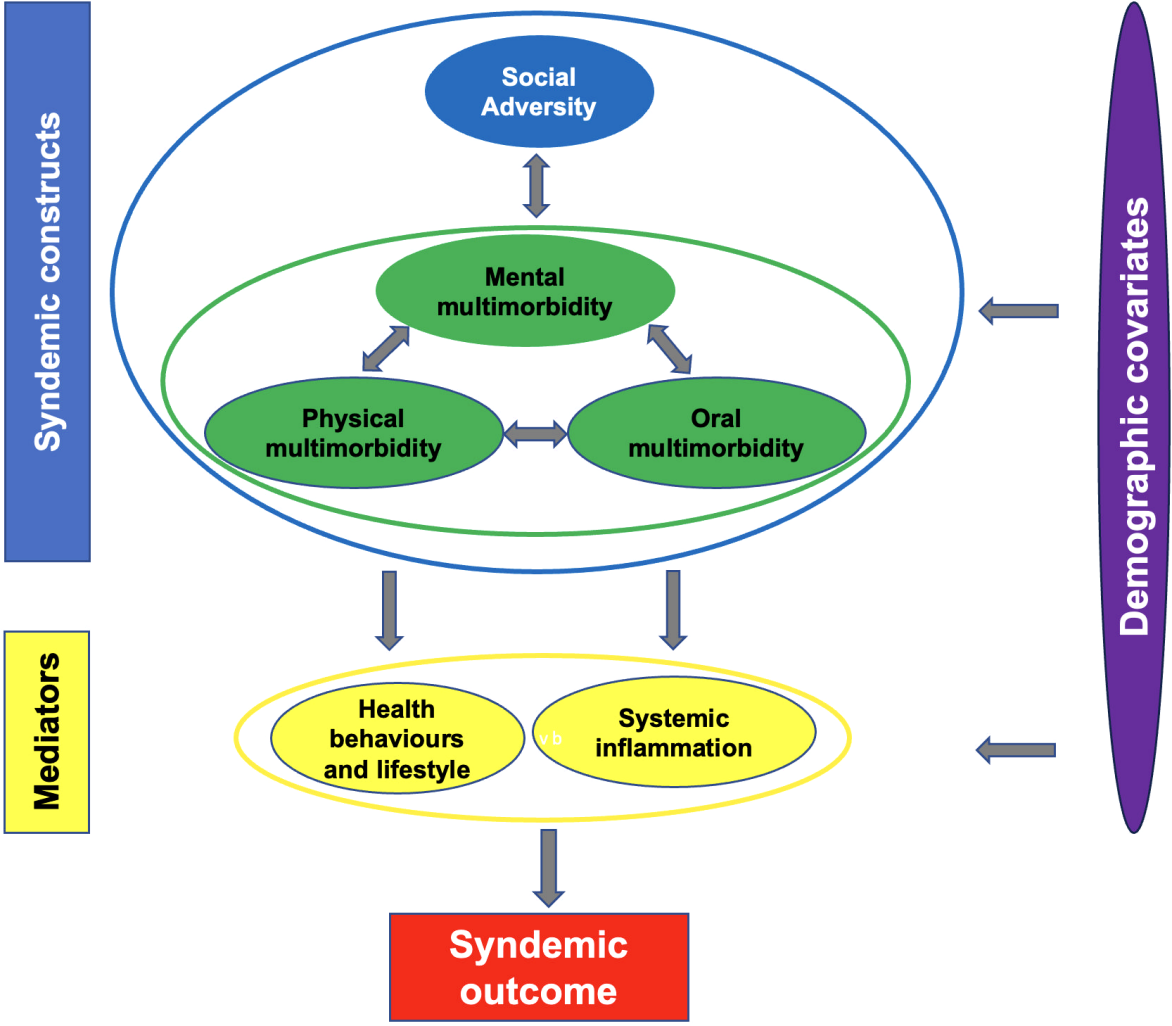
Theoretical framework to test for syndemic interactions between social adversity and mental, physical and oral multimorbidity.

Social adversity precipitates and exacerbates clusters of mental and physical diseases and conditions (**Figure 1**). For example, socioeconomic adversity (such as having low income, being unemployed or living in a deprived area) and traumatic events (such as adverse childhood experiences, being a victim of physically violent crime or sexual assault) are linked to compounded mental and physical conditions via systemic inflammation and behavioural pathways (10, 11). The situation can be further compounded by barriers to accessing healthcare (12). For instance, people with severe mental illness may experience under-recognition and under-treatment of physical conditions such as cardiovascular disease (12–14). In turn, these clustering mental and physical conditions reinforce each other through bidirectional interactions, that are also shaped by the social and contextual factors, leading to multiplicated mortality and morbidity burdens (8).

Oral diseases could be a key element in the above social adversity, mental and physical syndemic. They share the same social determinants and have bidirectional interactions with mental and physical conditions, precipitating and perpetuating their personal, social and economic impacts (1, 15). For example, the higher rates of oral diseases and conditions, such as dental caries, periodontal disease and tooth loss, and the increased risk of the late detection of oral cancer in people with mental disorders compared to the general population are alarming (16–18). Poor oral health affects simple daily functions such as eating, smiling and socialising, and increases social stigma and limited employment opportunities experienced by people with mental disorders (19–21). Oral conditions were the 3^rd^ most common reason for preventable hospital admissions in people living with severe mental illness (22). Indeed, people with mental disorders considered improving their oral health as part of bouncing back to normality (23).

Singer et al. underscored the importance of conducting further studies to explore new syndemics (8). Zahid et al. called for future studies to explore further specific potential syndemics involving mental disorders (24). No previous study has investigated the potential syndemic relationship between social adversity and mental, physical and oral multimorbidity (1, 25). Thus, the primary aim of the present work is to test for syndemic interactions between social adversity (socioeconomic adversity and traumatic events) and mental, physical and oral multimorbidity using the theoretical framework depicted in **Figure 1**. The secondary aim is to determine whether the syndemic relationships vary by age, sex and ethnicity.

## Methods

To achieve the above aims, data from three large-scale population-based databases: UK BioBank, US National Health and Nutrition Examination Survey (NHANES) and the Research with East London Adolescents Community Health Survey (RELACHS) will be used. The following summarises research questions and data analysis plan for each dataset.

### UK BioBank research questions

UK BioBank database will be used to answer the following research questions:

- Are there syndemic relationships between social adversity and mental, physical and oral multimorbidity impacting mortality burden and all-cause hospitalisation?
- Do these syndemic relationships vary by age, sex and ethnicity?

### UK BioBank methods

The present project’s methodology builds on published advances in testing the syndemic theory and personal communications with scholars working currently on testing the syndemic theory using UK BioBank data.

#### Design and study population

UK Biobank database comprises prospective biomedical data from 502,412 participants living across the UK and aged between 40 and 69 years at the time of recruitment (2006 to 2010). It benefits from data linkages to other health related records: primary care data, hospital admission data and cancer and death registries.

#### Syndemic outcomes

The syndemic outcomes are premature mortality and all-cause hospitalisation. Mortality data are obtained from the death register based on death certificates held within the NHS Digital (England and Wales) and the NHS Central Register (Scotland) between 01/01/2011 and 30/09/2021. All-cause hospitalisation data are obtained from the Hospital Episode Statistics data - Admitted Patient Care (England), Scottish Morbidity Records – General / Acute Inpatient and Day Case Admissions (Scotland), and Patient Episode Database (Wales) between 01/01/2011 and 28/02/2018.

#### Syndemic constructs

Syndemic constructs include social adversity (socioeconomic adversity and traumatic events) and mental, physical and oral multimorbidity.

Socioeconomic adversity will be measured by socioeconomic position (SEP) indicators at individual, household and neighbourhood (area) levels: education, employment, income and deprivation. Education is measured at an individual level and will be categorised into high (college/vocational training or above) and low (A levels, AS levels, or equivalent; O levels, GCSEs, or equivalent; CSEs or equivalent; NVQ, HND, HNC, or equivalent; other professional qualifications; and “none of the above”). Employment status is measured at an individual level and will be grouped into: employed (including those in paid employment or self-employed, or doing unpaid or voluntary work) and unemployed (including being retired, full or part time students, unemployed, looking after home and/or family, or unable to work because of sickness or disability). Income is measured by the annual total household income before taxes are deducted. It will be categorised into 0 (>£100,000), 1 (£52,000-£100,000), 3 (£31,000-£51,999), 4 (£18,000-£30,999), and 5 (<£18,000). Area deprivation is measured by Townsend deprivation index quintiles. The two quintiles with the highest area deprivation scores will be scored 1 (indicating high levels of area deprivation), and the rest of quintiles will be scored 0 (indicating low levels of area deprivation).

Traumatic events will include felt hated by family member as a child, did not feel loved as a child, physically abused by family as a child, sexually molested as a child, no one to take to doctor when needed as a child, victim of physically violent crime, victim of sexual assault, witnessed sudden violent death, belittlement by partner or ex-partner as an adult, physical violence by partner or ex-partner as an adult, sexual interference by partner or ex-partner without consent as an adult, and been involved in combat or exposed to war-zone.

Mental multimorbidity will include mood disorders and severe mental illness (ICD-10 codes: F20-F29; F31; F33-34).

Physical multimorbidity will be measured using Ronaldson et al. approach that comprises a total of 36 long term physical health conditions (**Supplementary Table 1**) (22).

Oral multimorbidity will include the presence of moderated to severe periodontal disease, toothache and use of denture. Self-reported painful gums and loose teeth will be used as surrogates for moderate to severe periodontal disease. In cases of multiple responses, the most severe indicator will be used as the primary surrogate for periodontal disease. Participants who did not report the above oral health indicators will be considered periodontally healthy.

#### Mediators

Mediators are health behaviours and lifestyle, and C reactive protein (as an indicator of systemic inflammation).

Health behaviours and lifestyle variables will include obesity (BMI>=30), alcohol intake (healthy versus unhealthy level; the former is defined as daily consumption of two drinks or fewer for men and women, according to the dietary guidelines in the UK - one drink contains 8g in the UK), smoking (healthy versus unhealthy levels; the former is defined as never smoked or smoking fewer than 100 cigarettes in life), physical activity (healthy versus unhealthy; the former is defined as 150 minutes of moderate-intensity physical activity weekly or 75 minutes of high-intensity physical activity weekly), poor appetite or overeating (Yes versus No), diet quality (healthy versus unhealthy level; the former is defined as meeting at least five out of the following ten dietary recommendations: 3 servings/day of fruit, 3 servings/day of vegetables, 3 servings/day of whole grains, ≥2 servings/week of (shell)fish, 2 servings/day of dairy, 2 servings/day of vegetable oils, ≤2 servings/day of refined grains, ≤1 serving/week of processed meats, ≤2 servings/week of unprocessed meats, and no sugar-sweetened beverages) (26, 27), trouble falling/staying asleep or sleeping too much (Yes versus No), ever addicted to any substance or behaviour (Yes versus No), and lifetime number of sexual partners.

The level of C-reactive protein (CRP) is used as a marker of bodily (systemic) inflammation and presented in mg/L.

#### Covariates

Covariates are demographic variables: age, sex and ethnicity.

### NHANES research questions

NHANES database will be used to answer the following research questions:

- Are there syndemic relationships between social adversity and mental, physical and oral multimorbidity impacting mortality burden and all-cause hospitalisation?
- Do these syndemic relationships vary by age, sex and ethnicity?

### NHANES methods

#### Design and study population

The NHANES program is conducted in 2-year cycles by the Centers for Disease Control and Prevention. Data are obtained from self-reported questionnaires, clinical examinations and lab tests. Each cycle includes nearly 10,000 participants and is representative of the United States population across all age groups. In this project, NHANES cycles between 2009 and 2014 will be used due to required data availability and consistency. Attempts to harmonise variables’ selection across NHANES and UK BioBank databases are made.

#### Syndemic outcomes

The syndemic outcomes are premature mortality and all-cause hospitalisation. Mortality data are obtained from the National Death Index (available up to 31 December 2015). All-cause hospitalisation data are based on being hospitalised in the year preceding the survey.

#### Syndemic constructs

Syndemic constructs include social adversity (socioeconomic adversity) and mental, physical and oral multimorbidity. No data is available in NHANES on traumatic events.

Socioeconomic adversity will be measured by SEP indicators at individual and household levels: education, employment, occupation, income and health insurance. Education is measured at an individual level and will be categorised into high (college or above) and low (high school diploma or below). Employment status is measured at an individual level and will be grouped into: employed (working at a job or business or with a job or business but not at work) and unemployed (looking for work or not working at a job or business). Occupation is measured using socioeconomic index, which is based on the employees’ earnings, education level and prestige. It will be categorised based on the median into upper and lower level and unemployment. The lower level will include retirees and students. Income is measured by family income ratio to the federal poverty line (<100%, 100%-199%, 200%-399%, >400%). Health insurance will be categorised into private health insurance (including any private health insurance, Medi-Gap or single-service plan), public health insurance only (including Medicare, Medicaid, State Children’s Healthcare Plan, military healthcare, Indian Health Service, State Sponsored Health Plan or other government programme) and no health insurance. No data is available in NHANES on area deprivation.

Mental multimorbidity will include depression (“moderately-severe” or “severe” depressive symptoms versus “moderate”, “mild” or “no” depressive symptoms; as measured by the PHQ-9) and severe mental illness (as measured by medicines prescribed in the past month for severe mental illness).

Physical multimorbidity will be measured using Ronaldson et al. approach (**Supplementary Table 1**) (28). However, not all the 36 long term physical health conditions are reported in NHANES (such as bronchiectasis, psoriasis and polycystic ovarian syndrome). Only those that are available in NHANES will be used to indicate the physical multimorbidity construct.

Oral multimorbidity will include the presence of moderate and severe periodontal disease, tooth loss (as a continuous and categorical variable: being edentate versus dentate), number of decayed teeth (as a continuous and categorical variable: having no decayed teeth versus having one or more decayed teeth), self-reported oral pain in the past year (“very often”, “fairly often” or “occasionally” versus “hardly ever” or “never”) and self-report global oral health (“excellent”, “very good” or “good” versus “fair” or “poor”). Periodontal disease (moderate and severe versus mild or no periodontal disease) will be measured by the clinical attachment loss (CAL) and periodontal probing depth (PPD). Severe periodontitis is defined as having 2 or more interproximal sites with CAL 6 mm or greater (not on the same tooth) and 1 or more interproximal sites with PPD 5 mm or greater. Moderate periodontitis is defined as 2 or more interproximal sites with CAL 4 mm or greater (not on the same tooth) or 2 or more interproximal sites with PPD 5 mm or greater (also not on the same tooth). Mild periodontitis is defined as 2 or more interproximal sites with CAL 3 mm or greater and 2 or more interproximal sites with PPD 4 mm or greater (not on the same tooth) or 1 or more sites with 5 mm or more.

#### Mediators

Mediators are health behaviours and lifestyle, and C reactive protein.

Health behaviours and lifestyle variables will include obesity (BMI>=30), alcohol intake (healthy versus unhealthy level; the former is defined as daily consumption of one drink or fewer for women and two drinks or fewer for men, according to the dietary guidelines in the US - one drink contains 14g of ethanol in the US), smoking (healthy versus unhealthy levels; the former is defined as never smoked or smoking fewer than 100 cigarettes in life), physical activity (healthy versus unhealthy; the former is defined as 150 minutes of moderate-intensity physical activity weekly or 75 minutes of high-intensity physical activity weekly), poor appetite or overeating (Yes versus No), diet quality (healthy versus unhealthy; the former is defined as the health eating index in the top two fifths of distribution), trouble falling/staying asleep or sleeping too much (Yes versus No), ever addicted to any substance or behaviour (Yes versus No), number of sexual partners in the past year, inconsistent condom use (“never” versus “occasionally” or “always”), toothbrushing (twice daily versus less than twice daily), dental flossing (once daily versus less than once daily) and dentist visiting (once every two years or more versus less than once every two years).

The level of CRP is used as a marker of bodily inflammation and presented in mg/L.

#### Covariates

Covariates are demographic variables: age, sex and ethnicity.

### RELACHS research questions

RELACHS database will be used to answer the following research questions:

- Are there syndemic relationships between social adversity and mental, physical and oral multimorbidity impacting self-reported general health?
- Do these syndemic relationships vary by age, sex and ethnicity?

### RELACHS methods

#### Design and study population

RELACHS is a 3-phase longitudinal school-based survey of a representative sample of a very ethnically and culturally diverse young people population residing in East London. Schools in the London boroughs of Hackney, Tower Hamlets and Newham were randomly selected and balanced to ensure representation of single and mixed sex schools. Children from Year 7 and Year 9 were recruited at baseline (time 1). Data from phase 1 (for syndemic constructs, mediators and covariates) and phase 3 (for the syndemic outcome) will be used. It is worth noting that oral health related data were only collected in phase 3.

#### Syndemic outcome

The syndemic outcome is self-reported health at phase 3 (very good, good versus fair, bad, very bad).

#### Syndemic constructs

Syndemic constructs include social adversity (socioeconomic adversity and adverse childhood experiences; ACEs) and mental, physical and oral multimorbidity.

Socioeconomic adversity will be measured by SEP indicators at individual, household and neighbourhood (area) levels: free school meals, car/van ownership, home ownership, parental (household) employment status and household overcrowding. Household employment status will be measured using the “dominance approach”, assigning the household the employment status of the parent/carer with the higher status.

ACEs will be measured by experiences of repeated bullying in school (“sometimes”, “about once a week” or “several times a week” versus “never bullied”, “I have not been bullied in school this term” or “once or twice”), lack of parent/carer involvement; experiences of parents often arguing or fighting; being looked after; continuing family money problems; close family dying; and home suffering damp, mould, vibration, noise from neighbours, cold, road traffic noise or dust.

Mental multimorbidity will include depression and having mental health problems. The former is measured by the Short Moods and Feelings Questionnaire (where a sore of ≥ 8 is indicative of depression). The latter is measured by the Strength and Difficulties Questionnaire (where a score of ≥ 17.5 is indicative of having mental health problems) (29).

Physical multimorbidity will be measured by having chronic illness (asthma, eczema, epilepsy, diabetes, hearing problems, eyesight problems, hay fever, chronic fatigue syndrome/ME or other chronic illness).

Oral multimorbidity will include the number of decayed teeth (as a continuous and categorical variable) and presence of toothache.

#### Mediators

Mediators are health behaviours and lifestyle. No data is available in RELACHS on C reactive protein or other inflammatory markers.

Health behaviours and lifestyle variables will include being overweight or obese (having BMI above the 85th percentile and 95th percentile, respectively); alcohol consumption (never tried versus tried alcohol or consumed alcohol in the past week), smoking (never smoked versus ever tried or regular smoker), drug use (never tried versus tried drugs: cannabis, glue/solvents/gas, ecstasy, crack, heroin, amphetamines, LSD, cocaine and/or khat), physical activity (active versus inactive, exercising more or less than twice a week for an hour), diet as measured by the frequency of consumption of breakfast, crisps/savoury snacks, sweets, biscuits, fried food and fizzy drinks (“never”, “hardly ever”, “1-2 days a week” versus “3-4 days a week” or “daily”) as well as daily consumed portions of fruits and vegetables (5 or more portions daily versus less than 5 portions daily or none); dieting behaviour as measured by ever dieted to lose weight (Yes versus No) as well as present attempts to diet to lose or gain weight (Yes versus No); and tooth brushing (twice daily versus less than twice daily).

#### Covariates

Covariates are demographic variables: age, sex and ethnicity.

#### Statistical analysis

Descriptive statistics will be used to summarise participants characteristics. For NHANES and RELACHS data, survey analysis procedures will be used to account for the sample weights, stratification and clustering of the complex sampling design to ensure representative estimates.

To answer the research questions on the presence of syndemic relationships, a structural equation modelling (SEM) technique will be utilised to conceptualise syndemic constructs and model complex relationships between directly observed and indirectly observed (latent) variables. The SEM technique has gained increasing popularity in syndemic research (30, 31). To use this technique, the following steps will be undertaken:

In step one, exploratory and confirmatory factor analyses will be used to identify the clustering nature and reduce the number of indicators (observed variables) under each theoretical construct. In step two, a SEM to predict premature mortality and all-cause hospitalisation, controlling for age, sex and ethnicity, will be run. Analysis will include identifying whether the indicators chosen to measure the latent constructs are acceptable. confirmatory factor analysis will be used to provide information on how indicator items measure underlying (latent) constructs. In step three of the analysis, SEM will be used to examine the direct and indirect relationships between the constructs. The goodness of fit (GoF) of the SEM model to the data will be assessed considering the indices of Comparative Fit Index (CFI) and Tucker-Lewis Index (TLI; where values >.90 indicate a good fit), root mean square error of approximation (RMSEA), and Standardised Root Mean Square Residual (SRMR; where value <.05 indicate a good fit). In step four, the synergistic (multiplicative) interactions between different latent constructs and measured variables will be tested using two-step estimation procedures (32).

To answer the question on whether syndemic relationships vary by age, sex and ethnicity, stratified analyses by age, sex and ethnicity will be performed.

Full Information Maximum Likelihood technique will be used as a pragmatic approach for data missing estimation for SEMs. This approach produces unbiased parameter estimates and standards errors (33). For UK BioBank and RELACHS datasets, stability of exposure data over time will be checked by calculating the intraclass correlation coefficients between baseline and follow up data.

## Discussion

The syndemic conceptualisation provides a valuable framework to understand health and illness, and hence to better design and deliver effective and cost-effective preventative and curative care to improve patient and population health. For example, the syndemic theory highlights the paramount importance of the simultaneous treatment of all interlocked conditions. It also offers a helpful perspective to understand the reduced treatment efficacies and increased treatment costs when any single condition is tackled in isolation of the other conditions and shared socioenvironmental determinants.

The syndemic theory underscores the importance of having a holistic management approach through addressing the social determinants that gave rise to these interacting diseases and exacerbated their burdens (34). This in turn has many implications at a clinical practice and policy level. Rather than viewing diseases in isolation from the context in which they occur, the syndemic concept renders preventative care and treatment more effective as it emphasises the importance of tackling contextual adversities and vulnerabilities (35). Adopting holistic syndemics management necessitates upstream measures, such as housing policy to address homelessness, education policy that ensures equitable access to quality education and future employment and fiscal measures. It also ensures simultaneous access to screening, treatments and behavioural public health interventions for the compounded conditions. At a more downstream level, syndemics management includes referring patients to social prescribing that could help address some of the social and economic exacerbators of syndemics, such as poor housing and unemployment.

The syndemic theory also highlights the need for new and innovative integrated care models founded on the concept of “task sharing”. Task sharing means delegating tasks that are often carried out by specialists to general healthcare professionals and workers with specific training on holistic health models. For example, rather than having a healthcare assistant for depression and another for diabetes, healthcare assistants could receive syndemic care training to provide an array of brief interventions on weight management, physical activity, diet, smoking cessation, oral hygiene and mouth cancer early detection. They could also, as part of task sharing, screen for the potential multiple clustering and interacting diseases and social conditions. Indeed, this approach was supported in the new NHS England guidance for improving the physical health in people living with severe mental illness (36). Although the guidance did not explicitly frame proposed holistic care model from a syndemic stance the proposed actions to enhance the physical care for people with severe mental illness is an exemplar of syndemics care. It also links with contemporary approach to healthcare service management through more integrated care, helpfully, services are increasingly being set up in a way that facilitates syndemic management (37, 38).

In the abovementioned guidance, healthcare professionals who deliver the physical health checks and follow up care for people with severe mental illness screen for a myriad of physical, behavioural and social conditions, such as blood pressure, blood sugar, blood cholesterol, diet, smoking, alcohol intake, cancer screening, oral health, sexual health, COVID-19 vaccination and social disadvantage. They also offer brief interventions and support navigating pathways and taking up follow-up care, including smoking cessation, physical activity, weight management, oral hygiene, mouth cancer early detection and social prescribing. Task sharing provides invaluable opportunities to tackle inequalities in accessing care needed to manage syndemics (38). With the growing tendency to task sharing as the way forward to provide syndemics care, a potential concern could be the lack of remuneration to the healthcare workers who are sharing such tasks. Thus, ensuring appropriate incentivising schemes is crucial to support a holistic care that could reduce the excessive burden of compounded diseases in socially disadvantaged populations (34).

Empirical evidence is needed to demonstrate statistically how the syndemic understanding of health and disease, and hence the syndemics care, can improve the effectiveness and cost-effectiveness of health systems, and hence health outcomes (34). Models of syndemics care should be evaluated robustly using randomised controlled trials conducted in real world settings.
